# Insecticide resistance in *Anopheles gambiae *from south-western Chad, Central Africa

**DOI:** 10.1186/1475-2875-7-192

**Published:** 2008-09-29

**Authors:** Clément Kerah-Hinzoumbé, Mallaye Péka, Philippe Nwane, Issa Donan-Gouni, Josiane Etang, Albert Samè-Ekobo, Frédéric Simard

**Affiliations:** 1 Programme National de Lutte contre le Paludisme, N'Djaména, Tchad; 2 Organisation de Coordination pour la lutte contre les Endémies en Afrique Centrale (OCEAC), Yaoundé, Cameroun; 3 Université de Yaoundé 1, Yaoundé, Cameroun; 4 Division de l'Hygiène du Milieu et de l'Assainissement, N'Djaména, Tchad; 5 Faculté de Médecine et de Sciences Pharmaceutiques, Douala, Cameroun; 6 Institut de Recherche pour le Développement (IRD), UR016, Bobo-Dioulasso, Burkina Faso

## Abstract

**Background:**

Indoor residual spraying and insecticide-treated nets (ITN) are essential components of malaria vector control in Africa. Pyrethroids are the only recommended compounds for nets treatment because they are fast-acting insecticides with low mammalian toxicity. However, there is growing concern that pyrethroid resistance may threaten the sustainability of ITN scaling-up programmes. Here, insecticide susceptibility was investigated in *Anopheles gambiae *sensu lato from an area of large scale ITN distribution programme in south-western Chad.

**Methods:**

Susceptibility to 4% DDT, 0.05% deltamethrin, 0.75% permethrin, 0.1% bendiocarb and 5% malathion was assessed using the WHO standard procedures for adult mosquitoes. Tests were carried out with two to four days-old, non-engorged female mosquitoes. The *An. gambiae *Kisumu strain was used as a reference. Knockdown effect was recorded every 5 min and mortality scored 24 h after exposure. Mosquitoes were identified to species and molecular form by PCR-RFLP and genotypes at the *kdr *locus were determined in surviving specimens by Hot Oligonucleotide Ligation Assay (HOLA).

**Results:**

During this survey, full susceptibility to malathion was recorded in all samples. Reduced susceptibility to bendiocarb (mortality rate of 96.1%) was found in one sample out of nine assayed. Increased tolerance to pyrethroids was detected in most samples (8/9) with mortality rates ranging from 70.2 to 96.6% for deltamethrin and from 26.7 to 96.3% for permethrin. Pyrethroid tolerance was not associated with a significant increase of knock-down times. *Anopheles arabiensis *was the predominant species of the *An. gambiae *complex in the study area, representing 75 to 100% of the samples. Screening for *kdr *mutations detected the L1014F mutation in 88.6% (N = 35) of surviving *An*. *gambiae *sensu stricto S form mosquitoes. All surviving *An. arabiensis *(N = 49) and M form *An*. *gambiae *s.s. (N = 1) carried the susceptible allele.

**Conclusion:**

This first investigation of malaria vector susceptibility to insecticides in Chad revealed variable levels of resistance to pyrethroid insecticides (permethrin and deltamethrin) in most *An*. *gambiae *s.l. populations. Resistance was associated with the L1014F *kdr *mutation in the S form of *An. gambiae *s.s.. Alternative mechanisms, probably of metabolic origin are involved in *An. arabiensis*. These results emphasize the crucial need for insecticide resistance monitoring and in-depth investigation of resistance mechanisms in malaria vectors in Chad. The impact of reduced susceptibility to pyrethroids on ITN efficacy should be further assessed.

## Background

Vector control is a major component of the global strategy for malaria control which aims to prevent parasite transmission mainly through interventions targeting adult anopheline vectors [1]. Ongoing strategies rely heavily on the use of safe and effective insecticides through indoor residual spraying (IRS) or insecticide-treated nets (ITNs). Successful implementation of these strategies requires sound knowledge of vectors distributions, biology and susceptibility to available insecticide compounds. Currently, the National Malaria Control Programme (NMCP) of Chad is promoting large scale use of ITNs as the main vector control tool but little is known on the vectors responsible for malaria transmission in the country. Back to the 1960's, thirteen anopheline species were recorded [2], with mosquitoes from the *Anopheles gambiae *complex, including *An. gambiae *s.s. and *Anopheles arabiensis *being the most abundant and widespread. However, these data have never been updated and susceptibility to insecticides used for net treatment has never been assessed in Chad.

In many African countries, *An. gambiae *s.l. is developing resistance to all classes of insecticides used for mosquito control. Among these, pyrethroids are the only option for nets treatment due to their relative safety for humans at low dosage, excito-repellent properties, rapid rate of knock-down and killing effects [3]. The emergence and rapid spread of pyrethroid resistance in *An. gambiae *complex populations may be a threat for the sustainability of malaria vector control activities across Africa. Comprehensive knowledge of the factors underlying resistance is needed for the implementation of efficient vector control programmes including resistance management strategies. This raises the need for countrywide and regular surveys for monitoring the insecticide susceptibility status of major vectors, detecting resistance genes and assessing their implications on vector control activities [4]. Anopheline mosquitoes exhibit two major mechanisms of pyrethroids resistance: increased insecticide detoxification (metabolic resistance) and target site insensitivity [5]. Metabolic resistance to pyrethroids is usually associated with increased oxidases and esterases activity [6] while target site insensitivity results from point mutations in the voltage-gated sodium channel gene [7,8]. The latter mechanism also termed *kdr *(knock-down resistance) induces cross resistance to DDT. Two alternative *kdr *mutations have been described in *An. gambiae *s.l. populations, resulting in either a leucine-phenylalanine (L1014F), or a leucine-serine (L1014S) mutation at amino acid position 1014 of the sodium channel [7,8]. The former is widely distributed in the M and S forms of *An. gambiae *in West Africa, and the latter, originally described from Kenya, has been recently found in the S form of *An. gambiae *from Central Africa (see [9] for a recent review).

Metabolic and target site resistance have been found alone or occurring together in many countries bordering Chad as Cameroon [10-12], Central African Republic [13], Nigeria [14-16] and Sudan [17,18]. Pyrethroid insecticides are commonly used in Chad for crop protection and, more recently, for malaria vector control through large-scale ITN distribution programmes (NMCP, unpublished reports). In such a context, the emergence and spread of pyrethroid resistance is extremely likely in Chad. The present survey was hence designed to investigate the susceptibility of *An. gambiae *s.l. to the four classes of insecticides used in public health. It was carried out in Western Chad, in three health districts where ITNs distribution programmes are being implemented.

## Methods

### Study area

**Figure 1:**
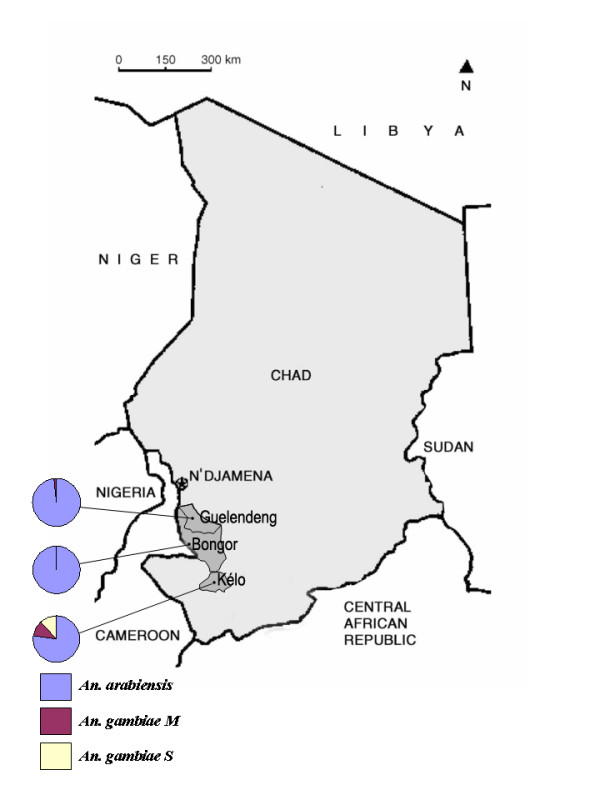
**Map of Chad showing the three health districts and their major city.** Pie chart give the relative proportion of members of the *Anopheles gambiae *in each district.

The study was carried out in August and September 2006 in the health districts of Bongor, Guelendeng and Kélo (Figure 1), where programmes for scaling up the use of ITNs are being implemented through mass treatment of nets available in the community and distribution of Long-Lasting Insecticidal Nets (LLINs) to children under one year of age and to pregnant women during immunization campaigns. The health district of Bongor lies along river Logone and is located in an area where rice cultivation is the main agricultural activity. Insecticide application is limited to individual use, often without respect of doses and frequencies of treatments, the products being purchased from small providers on the ground of informal advices, availability of the product and price. Bongor belongs to the sudanian climatic domain with annual rainfall about 700 mm. Mosquitoes were sampled in Bongor city (10.28°N; 15.37°E) and in the rural villages of Goulmoun (10.39°N; 15.20°E) and Tchinfoko (10.22°N; 15.27°E). The health district of Kélo belongs to the same bio-climatic domain and is situated in a cotton-growing area with a long tradition of insecticide use under supervision of the Government Agency for Agriculture Development. The mean annual rainfall in Kélo is about 800 mm. Mosquitoes were sampled in Kolon (9.24°N; 15.59°E), Bologo (9.08°N; 15.48°E) and Kélo city (9.19°N; 15.48°E). The health district of Guelendeng belongs to a transitional sudano-sahelian climatic domain with mean annual rainfall about 600 mm. The pattern of agricultural use of insecticide is the same as in Bongor but insecticides are only used for market gardening. Mosquitoes were sampled in Mbéré (10.47°N; 15.46°E), Witi-Witi (10.57°N; 15.32°E) and Guelendeng city (10.56°N; 15.32°E).

### Mosquito sampling

Wild *Anopheles gambiae *s.l. mosquitoes were collected as larvae from a range of breeding sites representative of the diversity of productive anophelines development sites in each locality, including puddles, foot and hoof prints on pond margins, shallow wells, tire tracks and rice fields. Larval collections were conducted in each of the nine localities during the raining season 2006. To limit consanguinity within samples, no more than 30 larvae (any instars) were collected per larval development site. All larvae from each location were then pooled together and reared locally until emergence. Emerging adults were provided with a 10% sugar solution. Adult mosquitoes were sexed and identified morphologically [2] and only females *An. gambiae *s.l. were used for insecticide susceptibility tests.

### Insecticide susceptibility tests

Insecticide susceptibility tests were performed using the WHO standard procedures and test kits for adult mosquitoes [19]. Impregnated papers with recommended diagnostic concentrations of 4% DDT, 0.05% deltamethrin, 0.75% permethrin, 0.1% bendiocarb and 5% malathion were purchased from the Vector Control Research Unit, University Sains, Malaysia [19]. Tests were carried out with two to four days-old, non engorged female mosquitoes. For each insecticide, 4 batches of 20 to 25 females were exposed to impregnated papers for 1 h. Control tests consisted of a group of 25 mosquitoes exposed to untreated papers. The *An. gambiae *Kisumu strain (provided by OCEAC, Yaoundé) was used as the reference strain and tested concomitantly. The number of knockdown mosquitoes were recorded every five minutes during the exposure period. Mosquitoes were then transferred into holding tubes and supplied with a 10% sugar solution. Mortality was recorded 24 h after exposure. The tests results were discarded if mortality in the control group was over 20%. If it was between 5 and 20%, mortality rates were corrected using Abbott's formula [20]. Dead and alive mosquitoes were kept separately in 1.5 ml tubes with silica gel and stored at -20°C for molecular analysis.

### Molecular identification and detection of the *kdr *mutations

For each village, a random sample of 40 mosquitoes drawn from the pool of unexposed mosquitoes (bioassays controls) considered as representative of the population being tested were identified using the PCR-RFLP technique described by Fanello *et al *[21] after DNA extraction according to Collins *et al *[22]. All mosquitoes surviving the bioassays were also identified in the same way and their genotype at the *kdr *locus was determined using the method of Lynd *et al *[23].

### Data analysis

The resistance/susceptibility status of the tested populations was determined for each insecticide according to WHO criteria. By the said criteria, a resistant population is defined by mortality rates less than 80% after the 24 h observation period while mortality rates greater than 98% are indicative of susceptible populations. Mortality rates between 80–98% suggest a possibility of resistance (suspected resistance) that requires confirmation. Times for 50% and 95% knockdown (KDT_50 _and KDT_95_) of tested mosquitoes were estimated using Win DL [24], a log-time probit software based on Finney [25].

## Results

### Susceptibility to insecticides

A total of 43 susceptibility tests were performed using 4% DDT, 0.05% deltamethrin, 0.75% permethrin, 0.1% bendiocarb and 5% malathion. Abott's formula was used to correct mortality rates of the Kisumu strain and the Mbéré sample to permethrin because mortalities in the control group were respectively 8.7 and 8%. No correction was required for the others populations, mortalities in control groups being always less than 5%.

The reference strain showed 100% mortality at diagnostic concentrations of deltamethrin, permethrin, bendiocarb and malathion. With 4% DDT, the mortality rate was 79.2%. Consequently, batches of impregnated papers with DDT were discarded from later use. All field collected *An. gambiae *s.l. were fully susceptible to malathion. Likewise, all tested populations were susceptible to bendiocarb, showing 100% mortality to this compound except in Mbéré where the mortality rate was 96.1%. Complete susceptibility to pyrethroids was only recorded in Guelendeng where mortality rates to deltamethrin (Table 1) and permethrin (Table 2) were respectively 98.9 and 100%. All other populations showed reduced susceptibility to both pyrethroids tested with mortality rates ranging from 70.2 to 96.6% for deltamethrin (Table 1) and from 26.7 to 96.3% for permethrin (Table 2). Resistance to deltamethrin was found in all villages in the Bongor district and in one village of the Kélo district (Kolon). Permethrin resistance was found in the three health districts, affecting all villages in the Bongor district, two villages in the Kélo district (Bologo, Kélo) and 1 village in the Guelendeng district (Mbéré).

**Table 1:** Knockdown times (KDT) and mortality rates of *An. gambiae *s.l. populations exposed to 0.05% deltamethrin

District	Village	N	Mortality (%)	% KD after 1 h exposure	KDT_50 _in min (95% CI)	KDT_95 _in min (95% CI)	KDT_50_R*
Bongor	Bongor	91	72.5^(3)^	93.4	27.5 (26.0–29.0)	72.5 (65.7–81.7)	1.70
	Goulmoun	85	72.9^(3)^	100	17.1 (16.1–18.1)	34.4 (32.0–37.5)	1.05
	Tchinfogo	94	70.2^(3)^	100	15.5 (14.7–16.3)	27.4 (25.6–29.7)	0.96

Guelendeng	Guelendeng	91	98.9^(1)^	100	13.4 (12.5–14.3)	29.0 (26.8–31.8)	0.83
	Mbéré	89	86.5^(2)^	100	11.2 (9.9–12.4)	20.4 (18.0–24.5)	0.69
	Witi-Witi	89	96.6^(2)^	100	9.5 (8.7–10.2)	20.3 (18.5–22.7)	0.59

Kélo	Kélo	100	88.0^(2)^	98.0	12.8 (11.8–13.7)	35.2 (32.3–38.9)	0.79
	Bologo	87	85,1^(2)^	96.6	25.2 (24.0–26.4)	50.6 (47.3–54.8)	1.56
	Kolon	99	74.8^(3)^	100	21.3 (20.4–22.2)	36.3 (34.3–38.7)	1.31

Kisumu Susceptible strain		82	100^(1)^	100	16.2 (14.6–17.7)	29.9 (26.7–34.9)	

* KDT_50_R: KDT_50 _of the tested population divided by KDT_50 _of the Kisumu reference strain (1): susceptible; (2): resistance suspected; (3): resistant (WHO, 1998)

**Table 2:** Knockdown times (KDT) and mortality rates of *An. gambiae *s.l. populations exposed to 0.75% permethrin

District	Village	N	Mortality (%)	% KD after 1 h exposure	KDT_50 _in min (95% CI)	KDT_95 _in min (95% CI)	KDT_50_R*
Bongor	Bongor	87	59.8^(3)^	87.4	35.1 (33.6–36.7)	74.1 (68.0–82.4)	2.17
	Goulmoun	90	26.7^(3)^	98.9	34.2 (33.1–35.3)	55.9 (53.1–59.6)	2.11
	Tchinfogo	94	48.9^(3)^	85.1	32.6 (30.7–34.4)	88.4 (78.2–103.6)	2.02

Guelendeng	Guelendeng	92	100^(1)^	100	14.6 (13.6–15.5)	27.3 (25.4–29.9)	0.9
	Mbéré	92	67.9^(3) ^**	98.9	16.9 (15.6–18.1)	38.2 (35.2–42.1)	1.04
	Witi-Witi	82	96.3^(2)^	100	18.3 (17.4–19.2)	31.1 (29.2–33.6)	1.13

Kélo	Kélo Urbain	97	76.3^(3)^	90.7	22.4 (20.2–24.5)	57.0 (49.9–67.8)	1.38
	Bologo	73	64.4^(3)^	94.5	24.8 (23.4–26.2)	54.8 (50.4–60.5)	1.54
	Kolon	88	89.8^(2)^	100	19.5 (18.5–20.5)	37.2 (34.8–40.2)	1.21

Kisumu Susceptible strain		100	100^(1) ^**	100	16.2 (15.2–17.1)	29.2 (27.3–31.7)	

* KDT_50_R: KDT_50 _of the tested population divided by KDT_50 _of the Kisumu reference strain (1): susceptible; (2): resistance suspected; (3): resistant (WHO, 1998)

** Corrected with Abott's formula

Pyrethroid resistance was not associated to a significant increase of knock-down times: in most cases, all mosquitoes were knocked down before the end of the exposure period, almost as quickly as the susceptible strain, even within resistant populations (Tables 1 and 2). This is examplified by the low values of KDT_50 _ratios and the overlapping of both KDT_50 _and KDT_95 _confidence intervals for the samples from several localities with those of the susceptible strain. Compared to that of the reference strain, a slight increase in KDT_50 _for deltamethrin was observed in Bongor, Bologo and Kolon. This increase in KDT_50 _was more noticeable with permethrin, affecting more populations, but it was only in the district of Bongor where the ratio was slightly over 2.0.

### Mosquito species, molecular forms and the *kdr *mutation

Out of 360 *An. gambiae *s.l. from control (unexposed) samples examined by PCR, 357 were successfully identified to species and molecular forms (Table 3). All specimens from the health district of Bongor were *An. arabiensis*. The samples from the health districts of Guelendeng and Kélo comprised respectively 98.3 and 77.3% of *An. arabiensis*. Only the M form of *An. gambiae *s.s. was found in Guelendeng (N = 2), while collections from Kélo revealed a mixture of the two molecular forms. PCR examination of surviving mosquitoes revealed that all specimens from Guelendeng (N = 43) and Bongor (N = 225) were *An. arabiensis*. In Kélo, both species of the *An. gambiae *complex were found within survivor mosquitoes. Overall, among the 36 *An. gambiae *s.s. specimens that survived insecticide exposure, one was of the M molecular form and the others belonged to the S form. Molecular assays detected the L1014F *kdr *mutation in 31 out of these 35 S form mosquitoes, with *kdr *frequency (in survivors) ranging from 60 to 90.5%, depending on the village considered (Table 4). The L1014S *kdr *mutation was not detected in any specimen. All surviving *An. arabiensis *and the M form specimen carried the susceptible allele.

**Table 3:** Composition of the *Anopheles gambiae *complex among pools of unexposed mosquitoes.

District	Village	Number tested	Species*		Molecular forms (%)	
				
			*An. arabiensis* N (%)	*An. gambiae* N (%)	M N (%)	S N (%)
Bongor	Bongor	40	40 (100)	0	-	-
	Goulmoun	40	40 (100)	0	-	-
	Tchinfogo	40	39 (100)	0	-	-

Guelendeng	Guelendeng	40	39 (97.5)	1 (2.5)	1 (100)	0
	Mbéré	40	39 (97.5)	1 (2.5)	1 (100)	0
	Witi-Witi	40	39 (100)	0 (0.0)	0	0

Kélo	Kélo	40	30 (75.0)	10 (25.0)	7 (70.0)	3 (30.0)
	Bologo	40	31 (79.5)	8 (20.5)	2 (25.0)	6 (75.0)
	Kolon	40	31 (77.5)	9 (22.5)	4 (44.4)	5 (55.6)

* 3 specimens could not be identified (one each in Tchinfogo, Witi-Witi and Bologo).

**Table 4:** Distribution of the L1014F mutation in mosquitoes surviving pyrethroid insecticide exposure in Kélo district.

***kdr *genotype^a^**	**Kélo**		**Bologo**		**Kolon**			**Total**		
				
	*An. gambiae S* (n = 21)	*An. arabiensis* (n = 9)	*An. gambiae S* (n = 9)	*An. arabiensis* (n = 24)	*An. gambiae M* (n = 1)	*An. gambiae S* (n = 5)	*An. arabiensis* (n = 16)	*An. gambiae M* (n = 1)	*An. gambiae S* (n = 35)	*An. arabiensis* (n = 49)
SS	1	9	1	24	1	2	16	1	4	49
SR	2	0	1	0	0	0	0	0	3	0
RR	18	0	7	0	0	3	0	0	28	0
f(*Kdr*)^b^	90.5	0.0	83.3	0.0	0.0	60.0	0.0	0.0	84.3	0.0

^a ^SS: Homozygote for the 1014L susceptible (wild type) allele; RR: Homozygote for the L1014F resistant allele; RS: Heterozygote (1014L/L1014F).

^b ^Frequency of the L1014F mutation in mosquitoes that survived after 1 hour of exposure to pyrethroid insecticides.

## Discussion

This study revealed the existence of permethrin and deltamethrin resistance in several *An. gambiae *s.l. populations from south-western Chad. Full susceptibility to malathion and bendiocarb was recorded in all samples tested, except in Mbéré, a village of the health district of Guelendeng where some level of tolerance to bendiocarb was observed. Full susceptibility to all insecticides tested was only observed with the sample from the urban area of Guelendeng. These various levels of insecticide susceptibility may reflect differential insecticide selection pressure exerted on field mosquito populations. The health district of Kélo is situated in a cotton growing area with intensive use of pyrethroid and organophosphorous compounds. The situation is quite different in Guelendeng and Bongor since chemical crop protection is not common in these areas. In fact, cotton cultivation in these two districts have ceased since the civil unrest in 1979. Before this date, organochlorines including DDT and dieldrin were regularly sprayed in the cotton fields. These regions experienced also chemical treatment against locust in the seventies [26]. Dieldrin, DDT, HCH and pyrethroids have also been used in the irrigated rice fields in Bongor but due to a lack of archives, appropriate information regarding doses and frequencies of treatment is not available. Some of these insecticides may have persisted in the environment, leading to subsequent selection of various resistance mechanisms in vector populations. In many African countries, resistance to pyrethroids has been attributed to extensive use of these compounds in agriculture [27,28], resistance levels being more important in cotton cultivation areas than in others agricultural schemes [12,29,30]. This is consistent with pyrethroid resistance being detected in the cotton growing area of Kélo. However, increased tolerance to pyrethroids in Bongor and, to a lesser extent in Guelendeng suggests additional selection pressure. Insecticide use for vector control interventions and/or personal protection against nuisances has been suggested as an additional putative source of selective pressure for pyrethroid resistance in malaria vectors, especially in urban cities and irrigated areas [30-32]. Indeed, in Bongor and adjacent areas, in addition to the recently introduced ITNs, coils and bomb sprays are frequently used for personal protection [33]. Further studies involving social scientists, chemical ecologists and environmental biologists would be needed to document the amount, frequency and diversity of insecticides used in these areas in order to explore in greater details the putative selective pressures leading to the selection of insecticide resistance in malaria vectors.

*Anopheles arabiensis *was found to be the predominant species of the *An. gambiae *complex in the study area. It constituted nearly 100% of the samples in Guelendeng and Bongor where the mean annual rainfall is the lowest. *Anopheles gambiae *s.s. was more abundant southwards in Kélo, where its two molecular forms were found to occur together with *An. arabiensis*, the latter still being predominant whatever the village. The species distribution within the *An. gambiae *complex is consistent with literature data for whole sub-Saharan Africa [34,35].

One of the main findings of the present study is the first report of the L1014F *kdr *mutation in wild *An. gambiae *populations from Chad. The resistant allele was found only in the S molecular form of *An. gambiae *s.s. in all villages sampled in the health district of Kélo. This finding is of paramount importance given recent evidences that *kdr*-based resistance mechanisms can jeopardize the efficacy of ITNs and IRS [36,37]. However, the strength of the correlation between the genotype at the *kdr *locus and the expression of insecticide resistance has been shown to vary in different genetic backgrounds an under different ecological settings [38,39]. Integrated investigations, using a more complete range of methodologies which allow detection of target sites mutations along with exploration of metabolic detoxification should be implemented in order to provide a more comprehensive overview of the genetic bases and mechanisms responsible for the resistance phenotype detected in these mosquito populations. Currently, the development of more sensitive tools is underway, greatly facilitated by recent advances in genomics. Some of these tools have been successfully tested, leading to more comprehensive knowledge of the molecular mechanisms of metabolic resistance [40,41]. Nevertheless, this new report of the L1014F mutation in *An. gambiae *from Chad further witnesses the ongoing spread of *kdr *mutations in Africa [9].

Absence of the *kdr *mutations in all surviving *An. arabiensis *suggests alternative resistance mechanisms, probably of metabolic origin are at play. This finding is not surprising because although both *kdr *mutations have been documented to occur in *An. arabiensis*, they are usually very scarce and are found floating at much lower frequencies than in its sibling, *An. gambiae *[12,17,32,42,43]. Increased monooxygenase activities were reported to be associated with pyrethroid resistance in major malaria vectors including *An. arabiensis *in East and Central Africa [6,11,44]. More recently, some general mechanisms occurring through a set of constitutively over-expressed genes with ability to control oxidative stress and other broad metabolic disorders was suggested to act as a general defence mechanism against deltamethrin in *An. arabiensis *populations from a neighbouring area of cotton cultivation in North Cameroon [12,41]. Similar mechanisms might occur in the *An. arabiensis *populations sampled in this study and further research into the mechanism(s) responsible for the high levels of resistance to pyrethroids occurring in western Chad are ongoing.

## Conclusion

This is the first investigation of malaria vectors susceptibility to insecticides in Chad. Variable levels of resistance to pyrethroids were found in most *An. gambiae *s.l. populations and the L1014F *kdr *mutation was detected in *An. gambiae *s.s. of the S molecular form. This finding provides additional evidence of the rapid spread of *kdr *mutations in *An. gambiae *throughout Africa. The mechanisms conferring pyrethroid tolerance in the sibling species, *An. arabiensis *need to be investigated. Pyrethroid resistance may seriously jeopardize the efficacy of IRS and ITNs on which most African countries, including Chad, rely to reduce malaria transmission. Careful monitoring of insecticide susceptibility and early reports of resistance in local malaria vectors are of considerable value for National Malaria Control Programmes, in order to properly devise and implement efficient and sustainable vector control strategies. Comprehensive implementation of vector control operations taking into account resistance management strategies is indeed required given the very few insecticidal compounds that are actually available for public health.

## Competing interests

The authors declare that they have no competing interests.

## Authors' contributions

CKH conceived and designed the study, coordinated its implementation in the fields, carried out laboratory procedures, analysed and interpreted data, and wrote the manuscript; MP carried out field experiments, analysed and interpreted data; PN helped with molecular processing, analysis and interpretation of data; IDG participated in the design of the study and supervised fields experiments; JE helped with data analysis and contributed in the drafting of the manuscript; ASE participated in the study design, participated in data analysis and interpretation and provided a critical review of the manuscript; FS participated in the study design, supervised fields and laboratory procedures, data analysis and interpretation, revised the manuscript and gave final approval for the version to be published. All authors read and approved the final manuscript.
